# Reproducibility Starts from You Today

**DOI:** 10.1016/j.patter.2020.100099

**Published:** 2020-09-11

**Authors:** Yasemin Turkyilmaz-van der Velden, Nicolas Dintzner, Marta Teperek

**Affiliations:** 1 Delft University of Technology, Mekelweg 5, 2628 CD Delft, the Netherlands

## Abstract

Who hasn't yet heard about the debates on research reproducibility, or, perhaps even more, about the research reproducibility crisis? There have been numerous papers in the past several years discussing reproducibility issues in research. In addition, funders, publishers, and research institutions followed policies aiming at increasing research reproducibility. But what does it mean in practice for research to be reproducible? And where does one start in this flood of information, tools, and requirements? In this article, we aim to help researchers improve the reproducibility of their work by providing simple tips and good practices that can be readily applied at different stages of the research life cycle. Reproducibility starts from you. Today!

## The Bigger Picture

In recent years, discussions about the reproducibility of scientific experiments have bloomed everywhere but have left researchers with either very high-level and unachievable goals or lost in a sea of recommendations. However, there are many small, simple steps that any researcher can take to improve the reproducibility of their results. Crucially, improving the reproducibility of one’s own research workflows offers numerous selfish benefits: not only making research more efficient (less time wasted!) but also increasing research impact and reach.

This article is aimed at researchers and offers several simple recommendations that can result in incremental improvements to the reproducibility of research results.

## Graphical Abstract

**Figure:**
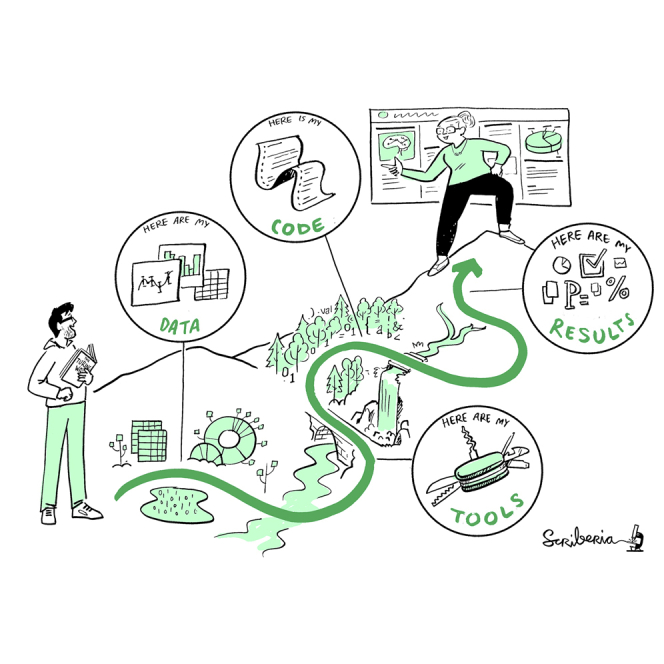
Reproducible Journey This image was created by Scriberia for The Turing Way Community^59^ and is used under a CC-BY license.

## Teaser

Research reproducibility has recently been gaining more attention, as can be seen in changes in funders' and publishers' policies regarding data availability and the emergence of the FAIR data principles. This has led to an increase in the number of requirements placed on researchers on how to handle, store, and archive their data. In this article, we aim to help researchers to work reproducibly by providing an overview of good practices at different stages of the research life cycle.

## Introduction

Ninety percent of 1,576 researchers who participated in *Nature*'s survey on research reproducibility agreed that there was a reproducibility crisis in science.^1^ According to this survey, up to 85% of the participants have failed to reproduce someone else's results, while up to 65% of the participants have failed to reproduce their own results. Respondents thought that intense competition, and practices such as “selective reporting” and “pressure to publish” were top contributors to irreproducible research. These were followed by factors such as “methods, code unavailable” and “raw data not available from original lab,” suggesting that sharing is not common practice in the current research culture.

Research reproducibility has been on the radar for some time^2^^,^^3^ and is certainly gaining more attention, as can be seen in changes in funders'^4^^,^^5^ and publishers'^6^^,^^7^ policies regarding data availability and the emergence of the FAIR data principles.^8^ FAIR data principles require data to be Findable, Accessible, Interoperable, and Reusable. The need for making these principles a requirement has been facilitated by the outcomes of various recent studies showing that data initially claimed to be available upon request are actually not available.^9^^,^^10^ This is not surprising given that in the current publishing culture the common practice is to disseminate research results by only publishing representative figures, or figures which are basically snapshots of finalized datasets, such as plots. These figures do not reveal the original data that has enabled their generation in the first place, nor do they explain how the data were analyzed. Additionally they are often not suitable for reuse, validation, and reproduction.

In this article, we provide some simple tips to help researchers increase the reproducibility of their research practices regardless of their domain. For the purpose of this article, we chose to use the term “reproducibility” to describe scientific activities aiming to obtain identical or similar results of a previously executed experimental or computational research activity in an identical or similar context. This article follows the life cycle of a research project. We start by outlining reproducibility concerns that should be addressed early in a project, followed by useful tools and practices to improve documentation and version control, and subsequently address the archiving of research outputs. We conclude with a discussion about reproducibility in a broader context.

## Planning for Reproducibility Should Start at the Very Beginning of the Project

Research reproducibility should be considered at the start of a research project. Otherwise, trying to establish all the connections between data, code, and the details of the methodology behind the experiments at the time of publishing papers describing the results might seem daunting if they have not been systematically recorded from the beginning.

So what are the tips for a good start?

### Carefully Design Your Research Project

Think about your research question and think about the methodology you will apply to answer the question. Think also about the data you will collect. How are you planning to collect these data, how will you analyze the data, and what kind of tools and infrastructure will you need?

### Write Up and Publish the Design of Your Study as a “Registered Report”

What this means is not only that someone will peer-review your study design and provide you with useful feedback, but you will also get a guarantee that the outcomes of your research will be published in a peer-reviewed journal, irrespective of the outcomes of your research. In other words, publishing your study design as a Registered Report^11^ really helps to tackle publication bias: all outcomes, “positive” and “negative,” are equally important for science to move forward. Publishing registered reports is a fairly recent practice but is already present in various domains such as humanities, medical research, and social sciences.^12^

### Start a Data Management Plan for Your Project

You might have heard about funders' requirements for data management plans (DMPs). If you get public funding for your research, a written DMP is likely to be one of the conditions of your grant. However, starting with a DMP is also an excellent practice by which to improve your research reproducibility. More and more research institutions now customize their guidance for writing a DMP to provide researchers with information about the most useful storage, backup, and version control solutions, as well as other tools and platforms available to them. Therefore, while writing a DMP, you will also make important decisions about where and how the data are stored during the project, and how to keep track of and version your data, which will all have important implications on the reproducibility of your research. In addition, when creating a DMP you will also obtain useful references to access ethics or data privacy resources, which might be essential for your project (e.g., do you need ethical approval? Do you need secure storage for confidential data?). Moreover, already at the planning stage, you can address strategies for long-term data preservation: what to keep, what not to keep, who will have access to the data, and how. How will you share your data after the project while respecting the ethical and legal regulations? A DMP can be seen as a roadmap with agreed data management practices for everyone involved in your research project. In other words, it is beneficial to start your project with a DMP whether it is required of you or not.

### Plan for Computational Reproducibility

Most of today's research includes some form of computation, often in the form of research software (from full-fledged applications to smaller analysis scripts). If software is an important component of your project, you should also consider how it will contribute to the overall reproducibility of your work. Common pitfalls in this area are missing dependencies (third-party software you use and forget to indicate in the documentation of your research software or analysis scripts), environment issues (operating system compatibility), and overly complex installation processes. The choice of software tools and platforms will have a pervasive effect on how you run your experiment and how you will share your results. It is, therefore, wise to consider those early in the planning phase.

There are a number of existing solutions to mitigate these issues and thereby make it easier for others to reproduce your work. Dependency managers, which are tools dedicated to software library dependency tracking and installation (MAVEN^13^ for Java or PIP^14^ for Python, among others), can facilitate the identification and/or download of libraries. Containers (such as Docker^15^) can ease the deployment of your tool. Containers allow you to work in an empty workspace, such that the execution of your software is not affected by everything that you previously installed on your computer. Although this forces you to document all your dependencies, you benefit from automated installation and execution facilities. If you are working on smaller projects, you might want to start with a notebook such as Jupyter Notebook.^16^ The advantage of using notebooks is that you can do everything in a single online environment: execute your scripts, and easily add notes and additional information. Using an online environment also means that you don't have to install anything—you start straight away. Most of these tools are open source, actively developed and supported, and commonly used in industrial settings. They all have extensive online documentation, with tutorials provided by the community.

### Ask for Help

This might all seem like quite a lot to do and feel intimidating, especially for those who are only starting with the whole reproducibility agenda. So don't hesitate to ask for help. Start with your peers. Perhaps they could provide you with useful tips on how to improve the reproducibility of your work, recommend some useful tools, or agree to review your code. Your university might also have dedicated resources to support you. More and more institutions employ dedicated disciplinary data stewards^17^ and data managers to advise researchers on data management practices, as well as research software engineers^18^ to provide professional programming support. In addition, academic libraries often have dedicated resources to help with data management and research reproducibility (advice, training, tools, and infrastructure). Even if community members are unable to help you directly, they will most likely connect you with someone who could.

## During Your Project: Documentation and Version Control Is Essential for Reproducibility

Documentation and version tracking of your experimental or computational protocols and setups are key for reproducibility. Your first setup may not be optimal, and you are likely to make adjustments to it. Keeping track of all these changes will save you a lot of time—you will always know what worked best and why.

### Document Your Experimental Work

If you are doing experimental work, it is important to write down all the details necessary for a future reader, a future colleague, and, most importantly, your future self to understand how you have carried out a certain experiment. There are various ways to do this. Typically, institutions require researchers to write everything down in a lab notebook. This can be done using a paper notebook; however, this makes it quite difficult to report details of data mostly captured in a digital setting. Also, what if your paper notebook gets accidentally lost or destroyed? Alternatively, you can use an electronic lab notebook (ELN).^19^^,^^20^ ELNs make it easy to digitally document your work, link your data and samples, record the parameters of experiments, or note down the results of any measurements. Moreover, ELNs are searchable and offer version control to make all your changes traceable. In addition to using an ELN, you can also add documentation about your data as a README.txt file (a plain text file) to the folder where the corresponding datasets are saved.^21^ Documentation may include details on the methodology used, analytical and procedural information, and data-specific information (such as parameters and/or variables, column headings, and codes/symbols).

### Document Your Computational Work

Understanding code is difficult, especially if you did not write it yourself. Documenting your code will help you and others understand how the code works, both for validation purposes and to build on the existing code base. To document your code, you can rely on the documentation standards of the programming language you are using. This can be done using, for example, “Docstrings” in R^22^ or Python,^23^ or Javadoc^24^ format in Java. Dedicated integrated software development (such as RStudio,^25^ Eclipse,^26^ or VS Code^27^) often facilitate the writing process of comments and provide plugins to generate documentation in PDF or HTML format.

There are many resources available online for such tool-supported practices.^28^, ^29^, ^30^ Therefore, by using an integrated development environment (most are free!), you can easily increase the reproducibility of your computation and facilitate long-term reuse.

### Use Version Control

**Figure 1:**
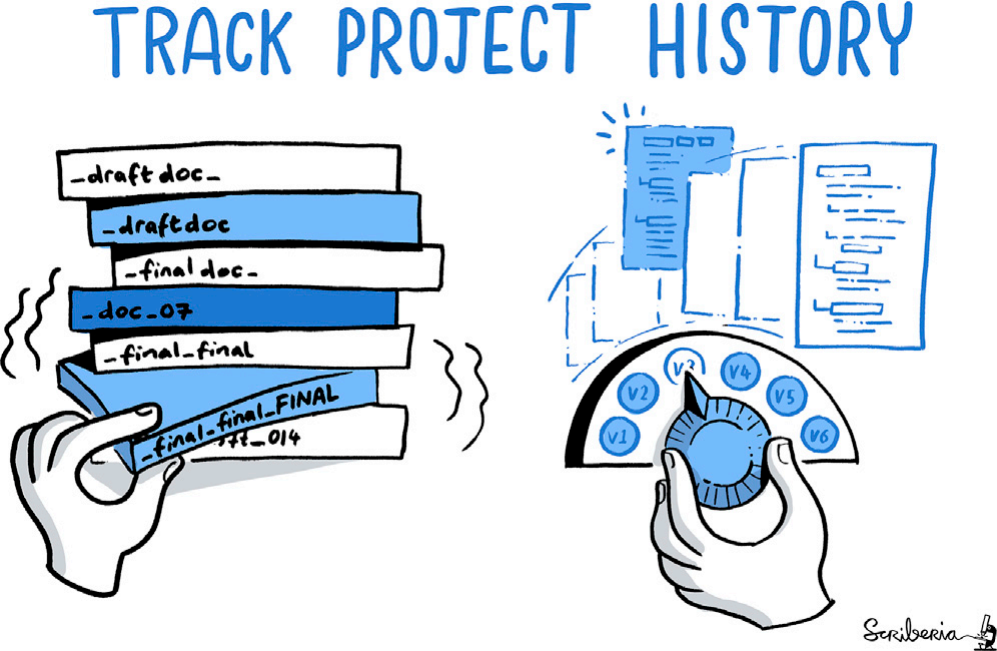
Project History This image was created by Scriberia for The Turing Way Community^35^ and is used under a CC-BY license.

Version control systems (such as Git,^31^ Mercurial,^32^ or Subversion^33^) help you keep track of all changes made but also who performed them. Such tools also allow easy tracking of the different versions of a given software program and facilitate the identification of specific valuable revisions (e.g., the version used in a specific publication), as illustrated in Figure 1. Widely used in industry, especially for source code management, these tools have great potential in a research context. You can use them for software source code as well as data. Version control will prove particularly useful if collaboration with multiple partners is expected. As version control tools have been used intensively for more than a decade, there are many online resources available, including “how to start” tutorials.^34^

## After Your Project: How to Share Your Work so that Others Can Reproduce It

Research takes a lot of time, money, and effort, and not every researcher has access to the same facilities. Therefore, sharing research outputs enables others with limited resources to analyze and reuse them for new purposes. Additionally, science can only progress if we can build on top of each other's results, and validation and reproduction of existing results is the first step.

Our first recommendation when it comes to archiving of research outputs is to consider the FAIR principles.^36^ The FAIR principles stand for Findability, Accessibility, Interoperability, and Reusability of research data. However, being FAIR is not a binary state, and one can strive to make their data “FAIRer.” In this section, we highlight what we consider to be critical for both long-term archiving and the FAIRness of research outputs while offering the most tangible selfish benefits.

So, how to share your work?

### Archive Your Outputs

Instead of sharing upon request you can archive your outputs in a repository, which is a place where things can be stored and shared. There are repositories for archiving datasets, protocols, software, and other outputs. You can find an overview of available repositories for data archiving in re3data.org^37^ or FAIRsharing.^38^ Such platforms index both domain dedicated repositories (such as Gene Expression Omnibus,^39^ NOMAD Repository,^40^ and PANGAEA^41^) and generic repositories, suitable for all domains (such as Zenodo^42^ or figshare^43^).

### Increase Your Citations

Perhaps the most important aspect of publishing outputs in a dedicated repository is obtaining a unique persistent identifier such as a Digital Object Identifier (DOI). This ensures that the archived outputs are always available online and prevents “404 not found” messages. Additionally, many repositories nowadays provide citation statements for the archived outputs, similar to academic papers. In this way you can get citations not only for your papers but also for the underlying outputs. If you are an early career researcher, it may take some time to build up a nice publication list. By publishing all your outputs, you can not only share the full breadth of your research (not just the snapshot presented in the research article) but also get more credit for your hard work and acquire increased visibility and impact.

In addition, you can also obtain a persistent identifier for yourself as a researcher. If you sign up for an ORCID iD^44^ (entirely free and not dependent on your research institution), you can ensure that all your outputs are linked to you and collected in one location, regardless of changes in your surname, email address, or affiliation.

### Get a License

Additionally, repositories offer a selection of licenses. By adding a license, you can make it clear to future users what they are allowed to do with your outputs and whether there are any restrictions on reuse. In general, Creative Common Licenses are suitable for data, while MIT, BSD, or Apache licenses are suitable for software. Choosing a license should be done with care.^45^ If in doubt, you should discuss this with all stakeholders involved in your research project and ask for support from the appropriate legal department. Be aware that without a clear license, data and code are protected by basic copyright laws, which are different in each country. Therefore, reusing data or code that does not have a license may make redistribution of your work (or part of it) more difficult.

### Share Your Protocols

Many research projects start with reproducing the outcomes of an existing study with the aim of building on top of it. However, the information provided in the Materials & Methods section of a publication is mostly limited, and statements such as “Contact the author for details” are not uncommon. As a result, many researchers spend months optimizing a protocol before they can get started with their project. You can share your detailed experimental and computational protocols on Protocols.io, which is a free and open platform.^46^ As you optimize your protocol, you can add new versions and link them to your original protocol. Each published protocol gets a DOI and can be linked to existing academic publications. In this way you can obtain citations for your protocols and save others months of work.

### Share Your Analysis Scripts and Research Software

Using a version control system is particularly crucial if you are developing analysis scripts and research software. Version control systems can be paired with online sharing platforms (such as GitLab,^47^ GitHub,^48^ or BitBucket^49^), making it even easier to collaborate, share, and archive your work. For example, there is a direct integration between GitHub and Zenodo, which is a general-purpose repository. By using the GitHub/Zenodo integration, you can get a DOI for your software and make it citable.^50^ Even if you do not use version control systems, you can always upload your code to Zenodo or another repository directly through their web interface.

## Open Aspects of the Reproducibility Discussion

We have provided you with multiple tips for carrying out reproducible research. However, it is worth stressing that the discussion about what exactly does it mean for research to be reproducible is ongoing. For example, researchers recently tried to reproduce 100 experiments in the field of psychological science. Researchers who participated in this study reflected that a reproduction experiment is but one experiment: “It is also too easy to conclude that a failure to reproduce a result means that the original evidence was a false positive.”^51^ This large-scale reproducibility study was quickly followed by remarks and comments^52^ on the statistical approach used to assess whether a replication attempt was successful or not. For instance, some suggested that the results of the reproducibility study actually showed a fairly high reproducibility rate. Camerer et al.^53^ further elaborated on the challenges of trying to reproduce reported results. The difficulty can come from testing multiple conclusions in a single paper, subtle changes in the experimental protocol, or differences in participant profiles in social science experiments. In addition, the statistical analysis approach used to estimate success or failure of the result of reproduction can also be questioned.

Therefore, the discussion about reproducibility is in a rather early phase. What are the criteria to judge whether a replication was successful? How many experiments do we need to consolidate results? “More” appears to be our current answer. The way science is executed might change in the future to accommodate for such observations. Working practices might need to be adjusted, as well as what is meant by reproducible research.

That said, you do not need to wait for this discussion to be over to start improving your working practices. There are easy ways to improve the reproducibility of your experiments.

## It Is All in Your Hands

There is a lot that can be done to improve research reproducibility, and there are two important things one should remember when thinking about it.

First, research reproducibility should be considered as a process. There are many steps that could be taken, and, as explained before, there is no single definition of “reproducible research.” So perhaps it is better to think about small steps and efforts that can be made today to make your research more reproducible. This should not be seen as a daunting task. Start small and build on as you go along.

Second, sometimes people wonder whose responsibility it should be to tackle the reproducibility crisis. Should we wait for funders to only fund reproducible research? Should we wait for the publishers to start publishing only reproducible papers? Should the institutions only hire and promote researchers who adhere to reproducible research practices? There are multiple stakeholders involved, all of whom could play a role in improving research reproducibility.

However, the key stakeholders involved, and at the same time the main beneficiaries, are the researchers themselves. So perhaps instead of thinking about whose responsibility it should be and who should take the first steps, it is better to consider the benefits of reproducible research practices. Florian Markowetz has written a useful article in *Genome Biology* with a suggestive title, “Five selfish reasons to work reproducibly”^54^ (and for those of you who would rather watch, than read, there is also a video recording of Florian's talk on the same topic^55^). These five reasons are:

• Reason 1: reproducibility helps to avoid disaster
• Reason 2: reproducibility makes it easier to write papers
• Reason 3: reproducibility helps reviewers see it your way
• Reason 4: reproducibility enables continuity of your work
• Reason 5: reproducibility helps to build your reputation

Thus, those who invest in improving the reproducibility of their research will be the first and foremost beneficiaries of these efforts.

So don't wait for the others to make a move, and instead think about what you can do today to improve the reproducibility of your own research practices. Every little step helps, and you will be the one who will benefit the most.

## Additional Resources

Finally, if you would like to learn more or join a community or an initiative aiming to improve research reproducibility, here is a list.

• The Turing Way: A Handbook for Reproducible Data Science^56^
• Software and Data Carpentry lessons:^29^^,^^30^ collaboratively developed and freely available lessons for teaching basic lab skills for research computing and universal data literacy
• Good enough practices in scientific computing:^57^ article about “good enough” practices that can be implemented immediately after a Software or Data Carpentry workshop
• ReproducibiliTea:^58^ a grassroots journal club initiative to discuss diverse issues, papers, and ideas about improving science, reproducibility, and the Open Science movement
• ReproHack:^59^ a hackathon to reproduce published research using publicly available associated code and data
